# Socioeconomic correlates, typology and characterization of indigenous guinea fowl (*Numida meleagris,* Linnaeus) farming in Benin, West Africa

**DOI:** 10.1016/j.heliyon.2022.e09226

**Published:** 2022-04-01

**Authors:** Boko Michel Orounladji, Folasade O. Oke, Koffi Tozo, Christophe A.A.M. Chrysostome

**Affiliations:** a Centre d’Excellence Régional sur les Sciences Aviaires, Université de Lomé, Togo; b Department of Agricultural Economics and Farm Management, Federal University of Agriculture, Nigeria; c Faculté des Sciences, Université de Lomé, Togo; d Faculté des Sciences Agronomiques, Université d’Abomey-Calavi, Benin; e Agricultural Media Resources and Extension Centre, Federal University of Agriculture, Nigeria

**Keywords:** Benin, Breeding system, Guinea fowl, Socio-economic, Typology

## Abstract

The role of agriculture in Benin, Western Africa cannot be overemphasized, where livestock production is a major occupation among the rural population as it serves as a means of livelihood and sustenance. This study was carried out to examine the socio-economic correlates of guinea fowl production status in Benin and to characterize their management practices. 165 farmers across 10 regions in Benin were selected using the non-probabilistic snowball sampling method. Information on farmers’ socio-economics, management practices and constraints to optimum production were elicited with the aid of a structured questionnaire and subjected to analysis. The results showed that across all the 10 regions surveyed, guinea fowl farming was the dominant occupation, particularly among men (81.0%) irrespective of sociolinguistic groups, religion and level of education. About one-third (34.5%) of the farmers had no formal education. In terms of characterization, four clusters of guinea fowl farmers were identified based on geographical location, educational level, management technique and farming experience. The constraints to guinea fowl production were slow growth and high mortalities as a result of diseases (40.7%) and predators (29.1%). Improvement in feeding (30%) and veterinary care (33.9%) were part of suggestions made by farmers to increase the productivity of the birds. The study concluded that adequate technical support and scientific research are inevitable in this sector as this will considerably improve the rural populations living conditions through enhanced income and therefore constitute a real lever for rural development.

## 1 Introduction

Like other countries in Sub-Saharan Africa where guinea fowl originated from, Benin, which is a country with a population of around 10 million people (INSAE, 2015), is also involved in indigenous guinea fowl production. Mishra et al. (2001); Sayila (2009) and Traore et al. (2018) in their various studies found that guinea fowls are generally more resistant than chicken and turkey to most of the common poultry viral diseases such as Newcastle disease, Fowl pox and Gumboro. Guinea fowls are also well adapted to traditional breeding production systems and as such occupy an important position among rural farming households (Mishra et al., 2001). Guinea fowl contributes immensely to rural households as it serves as a source of animal protein (meat and eggs), income generation from the sale of eggs and birds, and thus improving the food security condition and consequently poverty reduction among rural households (Kouassi et al., 2019). Guinea fowl meat and eggs are an excellent choice, both gastronomically and dietetically, with a high protein and low fat content (Lopez-Pedrouso et al., 2019). Apart from income generation and nutritional benefits, guinea fowl perform social and cultural roles in many African societies (Dahouda, 2003; Sanfo et al., 2009). Despite the socio-economic importance of guinea fowl in this region, it is faced with some challenges which hamper its optimum production among rural folks. Some of the challenges as documented by Traore et al. (2018) include a high rate of mortality particularly during the rainy season.

Furthermore, earlier studies (Boko et al., 2012; Houndonougbo et al., 2017) examined constraints to optimal poultry farming among households in the Sudanian and Sudano-Guinean areas of Benin. However, in the Guinean zone and selected districts in Sudanian and Sudano-Guinean zones, constraints on optimal guinea fowl production have received little attention from researchers. Also, as guinea fowl farming is gaining prominence across all the three climatic zones (Sudanian zone, Sudano-Guinean zone, and Guinean zone) in Benin and the scarcity of documented information about their socioeconomic correlates and characteristics across the country limit actions to improve and develop its production. The aim of this study, was to gather data on indigenous guinea fowl farming practices in Benin and generate information that could enhance intervention strategies to combat the constraints and consequently improve the productivity of guinea fowl in Benin.

## 2 Methods

### 2.1 Ethical approval

The manuscript does not contain clinical studies or patient data. Verbal informed consent was obtained before the interview of the farmers and they have consented to the submission of the results of this study to the journal.

### 2.2 Study area

**Figure 1:**
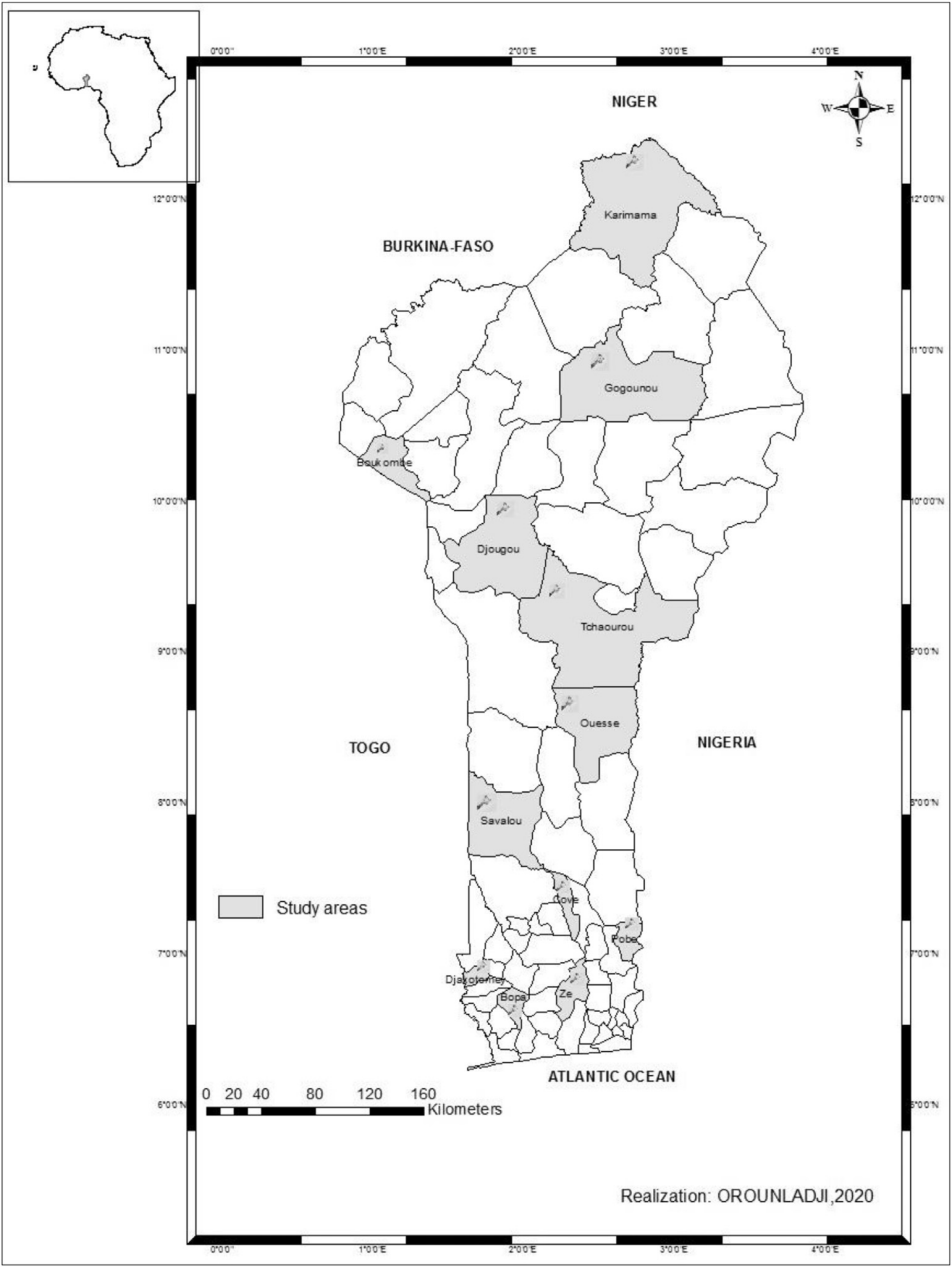
Map showing the studied areas.

The study was carried out within the entire Benin National territory. However, regarding the density of data collection, representative sampling was carried out to elicit information that could be taken into account to characterize the different rearing systems of indigenous guinea fowl among farmers in Benin. To this end, 12 districts (Figure 1) spread over 10 regions were sampled at an average rate of 3 villages per district. A total number of 165 farmers were interviewed for this study.

### 2.3 Sampling

Sampling was carried out using the non-probability snowball sampling technique. This is a method through which farmers were selected based on their network and not from a database sampling probability (Kone et al., 2018). The first farmer interviewed gave information about other guinea fowl farmers who in turn also provided information about the farmers they knew (Johnston and Sabin, 2010; Kouassi et al., 2019). The sampling continued in each village until the list of guinea fowl farmers in the study area was exhausted. The choice of districts and villages were made considering the population of guinea fowl farmers published by Countrystat and the animal production technicians of the territorial agricultural development agencies.

### 2.4 Data collection

The study was carried out during the second half of 2019. A cross-sectional survey was carried out in the district outlined in Figure 1. Primary data was obtained through the administration of a questionnaire to each of the 165 local guinea fowl farmers sampled who reared at least 10 birds (in the same physiological status) on their farm. Information on socio-economic characteristics of farmers, flock size, breeding and herd management technique as well as challenges against optimal production were elicited.

Interview guide in local language was furthered employed among farmers as the majority were illiterates. This made it easy to validate the responses obtained from the respondents. In term of reproductive parameters, the hatch rate was computed by asking farmers the number of eggs laid by the birds per laying season and the number of eggs hatched. The mortality rate at one week and three months after hatching were also obtained as a ratio of the total number of dead birds to the total numbers of birds available atone week and three months respectively.

### 2.5 Statistical analyses

The data collected was entered into Excel 2013, before being imported into the R software (R Core Team, 2019) for statistical analysis. Multiple Correspondence Analysis (MCA) was used to obtain a representation of farms in the form of projections of plans defined by the first-factor axis as adapted from Audigier et al. (2017). Based on the coordinates of the farms on the main factorial axis, an Ascending Hierarchical Classification (AHC) was used to group the farms according to their proximity to one another as adapted from Kouassi et al. (2019). Descriptive statistics (frequencies, percentages, means, standard deviation) were used to summarize socio-demographic characteristics as well as other quantitative variables of the poultry farms surveyed. Chi-square test was used to test whether there is a significant difference or not in the farmers’ socio-demographic characteristics (gender, education level, religion, secondary occupation) across regions and also if guinea fowl production indices differ across regions or not. Non-parametric Kruskall-Wallis test was used to compare the means of guinea fowl production indices across regions. The significance threshold adopted was 5%.

## 3 Results

### 3.1 Socioeconomic characteristics of guinea fowl farmers

Guinea fowl rearing in the study area was largely (81%) dominated by men while very few (19.0%) women were involved as shown in Table 1. In terms of educational qualification, about one-third (34.5%) of the sampled farmers were illiterate while 36.4%, 26.1% and 3.0% had primary, secondary and tertiary education respectively. In addition, Atacora and Alibori regions had more illiterate farmers than the remaining eight regions. The highly educated guinea fowls farmers were found in Collines, Atlantique, Mono and Zou regions.

**Table 1:** General characteristics of the farmers surveyed.

Characteristics		Regions										Pooled
		Alibori	Atacora	Atlantique	Borgou	Collines	Couffo	Donga	Mono	Plateau	Zou	
Gender (%)	Male	54.0	100	83.0	72.0	95.0	90.0	83.0	82.0	90.0	82.0	81.0
	Female	46.0	-	17.0	28.0	5.00	10.0	17.0	18.0	10.0	18.0	19.0
Education level (%)	Non-formal	67.9	86.7	11.1	27.8	-	20.0	33.3	17.6	40.0	29.4	34.5
	Primary	28.6	13.3	50.0	27.8	35.0	70.0	41.7	41.2	30.0	41.2	36.4
	Secondary	3.60	-	33.3	44.4	55.0	10.0	25.0	35.3	30.0	23.5	26.1
	Tertiary	-	-	5.60	-	10.0	-	-	5.90	-	5.90	3.00
Religion (%)	Christianity	-	-	100	11.1	100	100	8.30	100	50.0	82.4	52.7
	Islam	100	-	-	77.8	-	-	91.7	-	20.0	-	33.3
	Traditional	-	100	-	11.1	-	-	-	-	30.0	17.6	13.9
Secondary occupation (%)	Crop farming	57.1	13.3	27.8	16.7	10.0	60.0	25.0	47.1	20.0	17.6	30.3
	Arts and crafts	-	-	-	11.1	5.00	-	-	-	-	-	1.80
	Livestock farming	25.0	86.7	72.2	61.1	85.0	40.0	66.7	52.9	70.0	76.5	61.8
	Trading	17.9	-	-	11.1	-	-	8.30	-	10.0	5.90	6.10

There were no religious discrimination in the rearing of guinea fowl in the study area as it cut across all the religious groups in existence in the study area. However, it was prominent among christians than the other two religious groups.

Other livelihood activities than guinea fowl farming among the respondents include crop farming (30.3%), trading (6.1%) and art and craft (1.8%). Specifically, of all the regions surveyed, the Alibori region had the lowest number (25.0%) while Atacora had the highest number (86.7%) of guinea fowl farmers.

### 3.2 Age, farming experience and number of labour employed

Age, farming experience and the number of labour employed across the sampled farms were shown in Table 2. Farmers from the Atacora region had the highest mean age (55 ± 10 years old) than other regions. Also, guinea fowl farming experience in years was higher in this group (33 ± 13) than in all other regions. Farmers made use of more farm labour or workers in raising guinea fowl in the regions of Alibori, Atacora, Collines and Donga by than the remaining regions.

**Table 2:** Quantitative variables describing the poultry farmers surveyed.

Variables	Alibori	Atacora	Atlantique	Borgou	Collines	Couffo	Donga	Mono	Plateau	Zou	*p-value*
Age of farmer (years)	47 ± 8^b^	55 ± 10^a^	44 ± 7^b^	41 ± 7^b^	41 ± 6^b^	48 ± 5^b^	46 ± 10^b^	49 ± 8^ab^	46 ± 7^b^	41 ± 9^b^	0.00
Farming experience (years)	23 ± 13^b^	33 ± 13^a^	6.0 ± 3^c^	14 ± 8^c^	13 ± 9^c^	15 ± 8^c^	15 ± 10^c^	11 ± 6^c^	11 ± 6^c^	12 ± 7^c^	0.00
Number of employed labour	4.0 ± 1^a^	4.0 ± 2^a^	1.0 ± 1^b^	2.0 ± 1^ab^	4.0 ± 2^a^	3.0 ± 1^a^	4.0 ± 2^a^	2.0 ± 1^ab^	2.0 ± 1^ab^	2.0 ± 1^ab^	0.00

Note: ^a, b^ Means with unlike superscripts in the same row differ significantly *(p < 0.05)*.

### 3.3 Body weight, mortality of guinea fowl according to age groups and causes

The average weight of guinea fowl was 1.3 kg. The heaviest guinea fowl were found in Couffo and Donga regions with an average weight of 1.5 kg. The lowest weights (1.2kg) were observed in Atlantique, Collines, Mono and Plateau regions (Table 3).

**Table 3:** Body weight and mortality by age group and their causes.

Variables	Modalities	Alibori	Atacora	Atlantique	Borgou	Collines	Couffo	Donga	Mono	Plateau	Zou	All	χ^2^	*p-value*
Body weight		1.4 ± 0.2^b^	1.4 ± 0.2^b^	1.2 ± 0.3^d^	1.3 ± 0.1^c^	1.2 ± 0.2^d^	1.5 ± 0.2^a^	1.5 ± 0.2^a^	1.2 ± 0.3^d^	1.2 ± 0.2^d^	1.4 ± 0.2^b^	1.3 ± 0.2	-	0.0
Mortality rate	0–6 weeks	47.5	41.7	50.0	58.1	45.5	58.8	42.3	54.8	41.7	56.7	49.1	19	0.3
	6–16 weeks	37.3	38.9	41.7	35.5	31.8	17.6	30.8	41.9	41.7	40.0	36.5		
	>16 weeks	15.3	19.4	8.30	6.50	22.7	23.5	26.9	3.20	16.7	3.30	14.4		
Causes of mortality	Diseases	48.3	45.5	34.0	40.9	37.7	41.7	44.4	42.5	33.3	39.5	40.7	32	0.2
	Predators	24.1	21.2	24.5	31.8	32.1	20.8	18.5	40.0	33.3	39.5	29.1		
	Flights/Theft	19.0	30.3	32.1	9.10	22.6	20.8	33.3	15.0	30.0	16.3	22.2		
	Accidents	8.60	3.00	9.40	18.2	7.50	16.7	3.70	2.50	3.30	4.70	7.90		

Note: ^a, b,c,d^ Means with unlike superscripts in the same row differ significantly *(p < 0.05)*.

Almost half (49.1%) of the farmers interviewed reported that the mortality rate was much higher in guinea fowl from 0 to 6 weeks of age compared to other age groups with mortality lowest (14.4%) when they are 16 weeks and above as shown on Table 3. These mortalities were often due to several causes such as diseases (40.7%), predators (29.1%), as well as thefts and accidents constituting 22.2% and 7.9% respectively.

### 3.4 Feed and water administration

Guinea fowl diet was largely composed of cereals (corn, sorghum, millet, fonio and sometimes rice) + supplements (maggot and termites) (58.1%), kitchen scraps (21.2%), cereals only (11.4%), other feeds (5.1%) and complete feed purchased (4.2%). Atlantique region is the region where farmers use whole feed more (15%) to feed guinea fowl (Table 4).

**Table 4:** Feeding and watering.

Variables	Modalities	Alibori	Atacora	Atlantique	Borgou	Collines	Couffo	Donga	Mono	Plateau	Zou	All	χ^2^	*p-value*
Feeding	Full feed	0	0	15	0	6.9	6.2	6.2	0	0	0	4.2	47.62	0.10
	Only cereals	28.6	5.3	0	4.2	13.8	6.2	12.5	15	15.4	12.5	11.4		
	Cereals + supplement	51.4	73.7	45	70.8	55.2	56.2	56.2	70	61.5	58.3	58.1		
	Kitchen scraps	14.3	21.1	35	20.8	20.7	25	18.8	5	23.1	20.8	21.2		
	Other feed	5.7	0	5	4.2	3.4	6.2	6.2	10	0	8.3	5.1		
Watering	Running water	35.7	50	55.6	41.1	45	30	28.3	47.1	35	52.9	42.1	89.33	0.00
	Well water	60.7	13.3	38.9	38.9	35	50	46.7	35.3	25	29.4	37.3		
	Other sources	3.6	36.7	5.6	20	20	20	25	17.6	40	17.6	20.6		

The water administered to the birds as shown on Table 4 were obtained from several sources. Most (42.1%) of the farmers used running water (backwater, rain, water pools), 37.3% used well water (drilling) and 20.6% from other sources (pump, benin water corporation).

### 3.5 Selling price at maturity

The selling price of guinea fowl varied significantly (p < 0.05) depending on the period of the year and from one region to another (Table 5). Generally, an adult guinea fowl (at least 16 weeks old) attracted the highest selling price in the regions of Plateau (3,000 FCFA) followed by Mono (2,935 FCFA) and lowest in Atacora (2,460 FCFA) and Collines (2460 FCFA). However, during the festive periods (Christmas, New Year and Easter), the prices of these birds were more expensive in the Atlantique region (3,500 FCFA) with a difference of up to 725 FCFA. With the exception of the Atlantic region, the regions of Couffo (3,210 FCFA), Donga (3,325 FCFA), Mono (3,280 FCFA) and Plateau (3,310 FCFA) also had the highest prices during the holiday season.

**Table 5:** Selling price of animals in FCFA.

Variables	Alibori	Atacora	Atlantique	Borgou	Collines	Couffo	Donga	Mono	Plateau	Zou	*p-value*
Regular periods (1)	2620 ± 210^bcd^	2460 ± 200^d^	2780 ± 220^abc^	2520 ± 65^cd^	2460 ± 510^d^	2770 ± 170^abc^	2790 ± 320^ab^	2935 ± 200^a^	3000 ± 180^a^	2610 ± 130^bcd^	0.00
Festive period (2)	2800 ± 410^b^	2850 ± 200^b^	3500 ± 435^a^	2860 ± 245^b^	2795 ± 610^b^	3210 ± 220^a^	3325 ± 3310^a^	3280 ± 250^a^	3310 ± 210^a^	2950 ± 125^b^	
Deviation|1–2|	180 ± 285^c^	400 ± 190^bc^	725 ± 280^a^	340 ± 240^bc^	335 ± 240^bc^	440 ± 170^b^	535 ± 310^b^	340 ± 110^bc^	310 ± 110^bc^	335 ± 140^bc^	

Note: ^a, b^ Means with unlike superscripts in the same row differ significantly *(p < 0.05);* 1 USD = 554.91 F CFA.

### 3.6 Strategies for improvement

Notable areas for improvement to ensure optimum productivity as suggested by the farmers from the study in order of importance as shown in Table 6 include veterinary care (33.9%) feeding (30%), introduction of improved breeds of birds (10.8%) and management technique (10.6%).

**Table 6:** Areas for improvement.

Modalities	Alibori	Atacora	Atlantique	Borgou	Collines	Couffo	Donga	Mono	Plateau	Zou	All	χ^2^	*p-value*
Feeding	20.0	48.3	27.1	26.1	39.6	29.6	33.3	26.5	43.5	24.0	30.0	59.9	0.07
Veterinary care	40.0	51.7	25.0	26.1	41.7	33.3	33.3	26.5	43.5	28.0	33.9		
Hygiene	1.70	-	8.30	10.9	6.20	3.70	3.70	10.2	8.70	10.0	6.60		
Habitat	13.3	-	8.30	8.70	6.20	7.40	-	12.2	4.30	10.0	8.10		
Management technique	10.0	-	18.8	10.9	2.10	11.1	14.8	14.3	-	16.0	10.6		
Introduction of improved breeds of birds	15.0	-	12.5	17.4	4.20	14.8	14.8	10.2	-	12.0	10.8		

### 3.7 Reproductive performance of indigenous guinea fowl

Information elicited from the farmers showed that egg-laying frequency in guinea fowl was seasonal and was between April and October, which mostly coincided with the rainy season (Table 7). Sexual maturity, ranged from 6 to 7 months. In a production cycle, an average and matured guinea fowl lay eggs for a period of 26 and 30 weeks with an average bird laying an average of 71 ± 16 eggs. The incubation period in guinea fowl varied between 26 and 29 days for an average hatchability of 74%. After hatching, the average mortality rate for guinea fowl was 10% which could increase up to 22% at three months of age.

**Table 7:** Reproductive performance of local guinea fowl.

Reproductive parameters	Description
Sexual maturity (months)	6–7
Age of first laying (months)	6–8
Egg laying period	Rainy season (April–October)
Egg laying period during a season (weeks)	26–30
Average number of eggs laid (eggs/guinea fowl/season)	71 ± 16
Incubation time (days)	26–29
Hatch rate (%)	74 ± 17
Mortality rate 3 months after hatching (%)	22 ± 12
Mortality rate one week after outbreak (%)	10 ± 6

### 3.8 Characterization of guinea fowl production system

**Figure 2:**
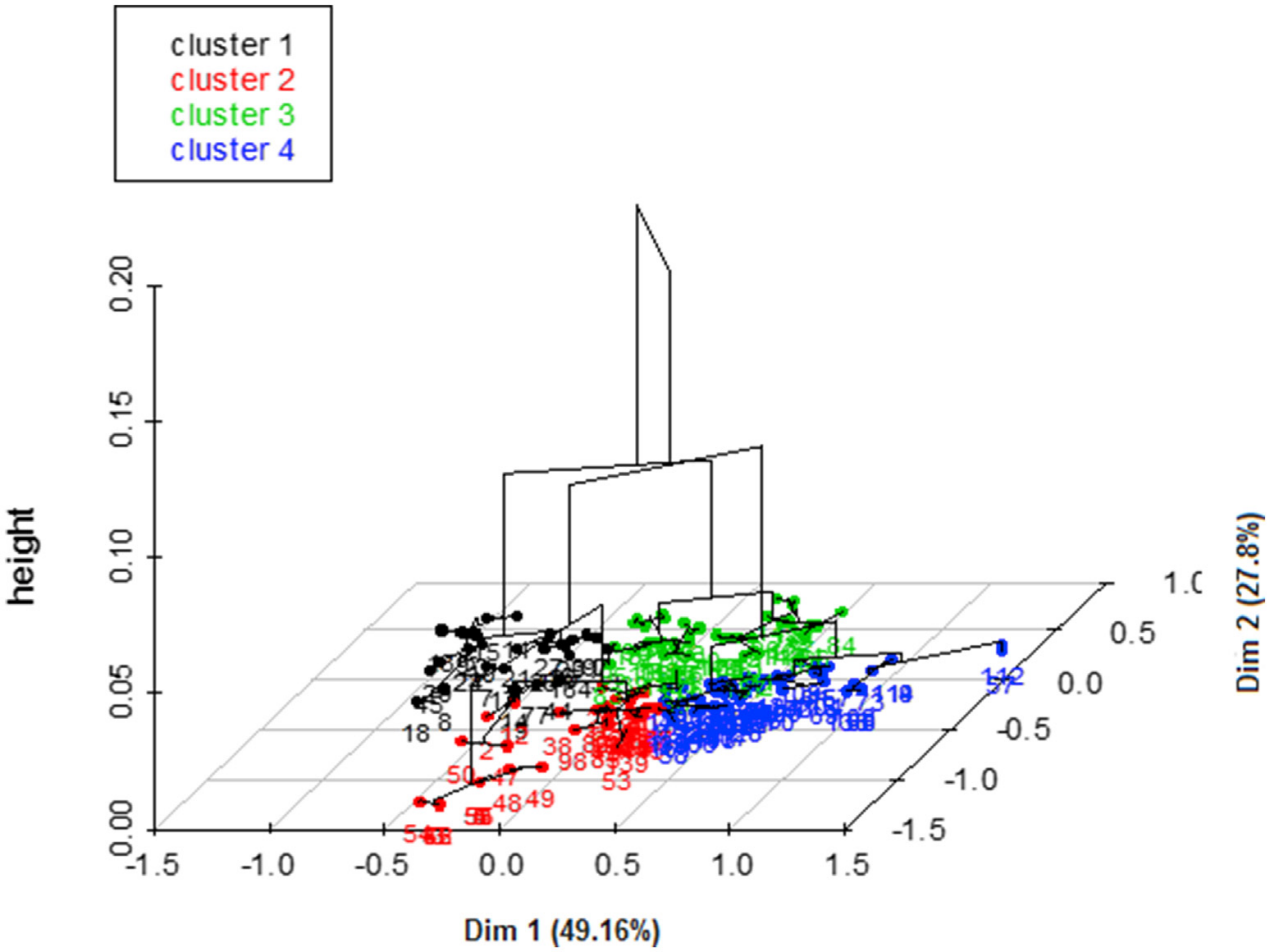
Projection of the ascending hierarchical classification on the factorial axes.

**Figure 3:**
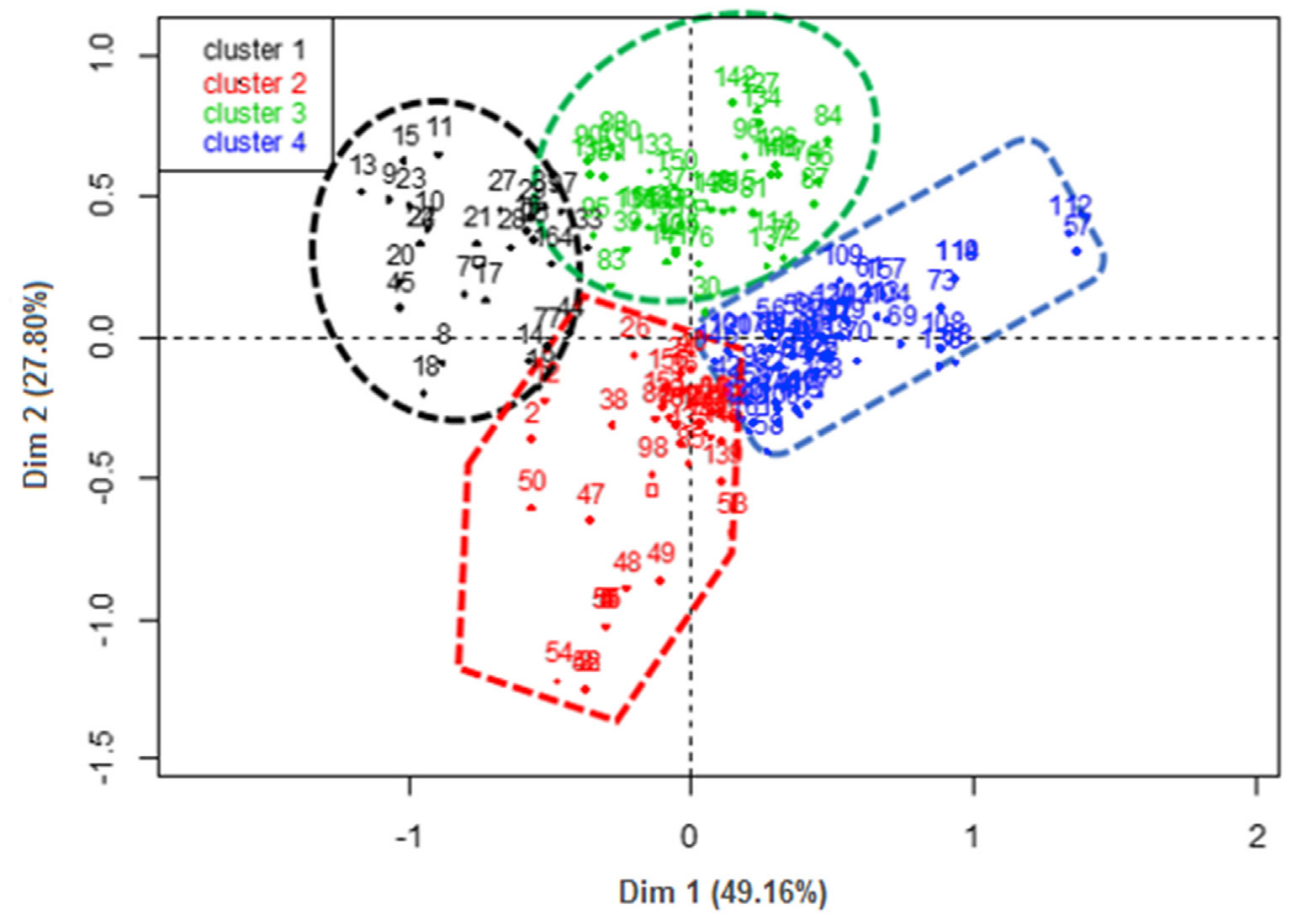
Projection of the poultry farms surveyed on the factorial axis.

Hierarchical clustering (Figure 2) was performed on 12 variables and this allowed four clusters to be retained (Figure 3). The cumulative contribution to the total inertia of the three selected factorial axis for hierarchical clustering was 92.88%. The frequencies of the different variables relating to the 4 categories of farmers are presented in Table 8.

**Table 8:** Characterization of Guinea fowl production System.

Characteristics		Cluster 1	Cluster 2	Cluster 3	Cluster 4	χ^2^	*p-value*
Geolocation of the farmer	Alibori	82.1	9.50	2.40	-	206	0.00
	Atacora	-	35.7	-	-		
	Atlantique	-	-	14.6	22.2		
	Borgou	3.60	7.10	22.0	9.30		
	Collines	-	2.40	7.30	29.6		
	Couffo	-	7.10	14.6	1.90		
	Donga	7.10	16.7	4.90	1.90		
	Mono	-	7.10	22.0	9.30		
	Plateau	3.60	9.50	4.90	5.60		
	Zou	3.60	4.80	7.30	20.4		
Gender of farmer	Male	50.0	95.2	70.7	92.6	30.0	0.00
	Female	50.0	4.80	29.3	7.40		
Age	Young (25–50 years)	57.1	50.0	80.5	77.8	17.6	0.01
	Old (51–60 years)	35.7	35.7	19.5	20.4		
	Very old (>60 years)	7.10	14.3	-	1.90		
Experience in guinea fowl breeding	Low (<5 years)	10.7	7.10	51.2	57.4	66.0	0.00
	Middle (5–10 years)	28.6	33.3	43.9	35.2		
	High (>10 years)	60.7	59.5	4.90	7.40		
Level of education	Illiterate	75.0	64.3	9.80	9.30	79.3	0.00
	Primary	17.9	33.3	51.2	37.0		
	Secondary	7.10	2.40	39.0	44.4		
	University	-	-	-	9.30		
Number of eggs at start-up	Low (<10 eggs)	39.3	16.7	26.8	16.7	23.72	0.00
	Middle (10–50 eggs)	60.7	83.3	58.5	59.3		
	High (>50 eggs)	-	-	14.6	24.1		
Farming system	Semi-intensive	39.3	69.0	78.0	72.2	16.71	0.01
	Intensive	3.60	2.40	7.30	3.70		
	Extensive	57.1	28.6	14.6	24.1		
Main activity	Crop farming	7.10	88.1	-	72.2	161	0.00
	Arts and crafts	-	7.10	2.40	13.0		
	Livestock farming	92.9	4.80	85.4	-		
	Trading	-	-	12.2	1.90		
	Public servant	-	-	-	13.0		
Secondary activity	Crop farming	64.3	4.80	73.2	-	148	0.00
	Arts and crafts	-	-	7.30	-		
	Livestock farming	7.10	95.2	14.6	100		
	Trading	28.6	-	4.90	-		
Incubation	Natural	100	100	78.0	83.3	15.6	0.00
	Artificial	-	-	22.0	16.7		
Additives in the feed	Product addition	-	-	7.30	5.60	4.79	0.19
	No addition	100	100	92.7	94.4		
Additives in water	Product addition	-	7.10	4.90	3.70	2.19	0.53
	No addition	100	92.9	95.1	96.3		

Cluster one was made up of 16.97% of the farmer sampled and were mainly (82.1%) located in the Alibori region. Most (75.0%) of the farmers were illiterate, that is, had no form of formal education and majority (60.7%) also had a minimum of 20 years experience in guinea fowl farming. The farmers in this cluster employed natural method of egg incubation and no additives were added to the birds’ feed and water to combat heat stress. The main and secondary activities of these farmers were livestock farming (92.9%) and crop farming (64.3%). Extensive method of farming was largely (57.1%) employed by farmers in this region.

Cluster 2 involved 25.45% of the farmers sampled. Most (59.5%) of the farmers had a minimum of 20 years farming and were located much more in the region of Atacora (35.7%) and Donga (16.7%). The farmers in this group were mostly (95.2%)men. They were mostly (64.3%) illiterate and relatively young, as their age ranged between 25 and 50 years (Table 8). The average number of eggs used in setting up their farm was between 10 and 50 eggs. In terms of heat stress management by farmers in this cluster, only 7.1% of the farmers used additives in the birds’ drinking water. Most (88.1%) of the farmers engaged in cropping as their main activity and animal husbandry (95.2%) as a secondary activity. Furthermore, 69% of these farmers had shelters for their birds to protect them from harsh weather condition and rest. This feature made them to rear their birds under a semi-intensive system of production. Eggs were also incubated naturally by farmers in this group.

Cluster 3 was made up of 24.85% of the farmers sampled. The farmers were mostly men (70.7%) and had primary education (51.2%). More than half (51.2%) of the farmers in this group had relatively little farming experience (<10 years) in guinea fowl production. Majority (85.4%) of these farmers engaged in animal production as their main activity whereas 73.2% made crop production their secondary activity. The majority of farmers in this group did not add any additive to the feed (92.7%) or to the water (95.1%) of guinea fowl to manage heat stress.

Cluster 4 in comparison to other groups, involved 32.73% of the farmers interviewed. These farmers were found mainly in the region of Collines (29.6%), Atlantique (22.2%) and Zou (20.4%). They were mostly male (92.6%) and between 25 and 50 years of age (77.8%). Very few (9.3%) had no formal education with 37%, 44.4 % and 9.3% each having primary, secondary and university education respectively. The production system was largely (72.2%) semi-intensive in nature with very few (3.7%) running an intensive system. Animal husbandry was essentially (100%) the secondary activity of farmers in this group while crop farming (72.2%) was their main activity. Artificial incubation was practiced by very few (16.7%) of the farmers.

## 4 Discussion

The predominance of men in guinea fowl production as deduced from the study may not be unconnected to the difficulty involved in managing guinea fowl due to the wild instinct that guinea fowl exhibits. This instinct, which is not very malleable constitutes a major constraint for the women in its production. This finding aligns with the earlier studies carried out in Ivory Coast (Kone et al., 2018; Kouassi et al., 2019); Burkina-Faso (Traore et al., 2018) and Ghana (Avornyo et al., 2016; Abdul-Rahman and Adu, 2017) that found that guinea fowl farmers were predominantly men. Similar findings were reported in Togo (Soara et al., 2020), Niger (Moussa Amadou et al., 2010) and Zimbabwe (Kusina et al., 2012). More attention on gender is required in policy design since women's engagement in integrated farming is undeniable. This same observation is common in most African countries. The study therefore emphasized the design and translation of technical manuals into local language due to the farmers' low level of formal education (Kone et al., 2018; Traore et al., 2018; Kouassi et al., 2019) as this will facilitate better understanding of the birds management requirement and subsequently lead to a sustainable development of this sector.

Guinea fowl farming however does not present any religious and cultural limits that could hinder its production in rural areas as deduced from the study. This agrees with the earlier submission of Kone et al. (2018) and Kouassi et al. (2019). Guinea fowl farmers engaged in other activities to diversify sources of income to meet up with the needs of their families. This result is in agreement with the findings of Traore et al. (2018). The age of the farmers were between 25 and 50 years which was similar to the earlier report of Traore et al. (2018) that young people under 20 did not have enough resources to go into guinea fowl production. Farmers received more support from their children which are the employees as family labor. To this end, these farmers take the opportunity to pass on their knowledge and prepare them to take over. Good experience must therefore be documented by reinforcing them with innovative practices to boost the sector to respond more effectively to food security within rural households and in response to climate change, as also evidenced by Fotsa (2008).

In the majority of farms surveyed, farmers preferred to use the local hen (natural incubation) to guinea fowl for incubating eggs due to their good brooding traits. The use of hens in villages for hatching guinea fowl eggs has been widely reported in other previous studies (Boko et al., 2012; Avornyo et al., 2016; Kone et al., 2018; Traore et al., 2018; Kouassi et al., 2019). In addition, guinea fowl and hen farming are closely linked (Boko et al., 2012). Most often, hens are used to incubate the guinea fowl's eggs. Sometimes farmers also make use of other poultry species such as duck and turkey for the incubation (Boko et al., 2012; Traore et al., 2018).

The feedstuffs used in formulating the diets of guinea fowl in Benin included corn, sorghum, millet, fonio and sometimes rice. These diets are supplemented in some farms with maggot and termites. This gesture of giving a few handfuls of these feed to the birds (guinea fowl) was done to train the birds so as to return to the farm after free-ranging. While on free-range, guinea fowl also feed on insects and grasses, which further diversified their diet. Traore et al. (2018) in Burkina Faso and Kouassi et al. (2019) in Ivory Coast, reported that the feedstuffs used for guinea fowl comprised sorghum, millet and corn. These results are different from those obtained in India (Gawande et al., 2007) where rice is the main diet offered to guinea fowl. These findings show that the choice of feed be offered to guinea fowl depends on the local availability and accessibility of the ingredients. The drinking water of guinea fowls was mostly non-drinkable. The lack of drinking water combined with the relentless distribution of mainly energy feed and kitchen waste to guinea fowl constitute factors that reduce the productivity of guinea fowl in rural areas. This same observation has been reported in Togo through the work of Lombo et al. (2018) who submitted that guinea fowl receive mainly energy feeds, thus affecting their productivity.

Guinea fowl farming was intended to provide additional income to households while providing them with low cholesterol meat (Abdul-Rahman and Adu, 2017). These households were also concerned about the sustainability of the sector by investing in the continuity of the flock. An earlier study reported that guinea fowl production contributes to the cultural and religious ceremonies of certain sociolinguistic groups, including the annual Ditamari festival (Dahouda, 2003). This study revealed that bonds of friendship and fraternity are strengthened through the use of guinea fowl as gifts to friends and also, in recognition of services rendered.

The large variation in the selling price of guinea fowl observed depended on the region and period of the year. In southern Benin, guinea fowl was more expensive due to the proximity to the urban centers and the low availability of the birds in this region. As for Northern Benin, the reluctance of farmers to sell guinea fowl in the rainy season because of it coincided with the reproduction period of these birds coupled with the higher demand of the birds during the festive period including Chrismas, New Year celebration and Easter, are the causes of the hike in the selling price of guinea fowl. The price increase ranged from 180 FCFA (0,324 USD) to 725 FCFA (1,307 USD) on the average. However, white guinea fowl were generally sold at higher prices than other phenotypes because they were in greater demand during religious ceremonies. Houndonougbo et al. (2017) also found that white guinea fowl had a higher selling price than those of other phenotypes of guinea fowl. Guinea fowl eggs were generally sold between 65 FCFA (0,117 USD) and 150 FCFA (0,270 USD).

The egg-laying period in guinea fowl was seasonal and lasted from April to October. During this period, mature guinea fowl were likely to lay an average of 71 ± 16 eggs distributed over 26–30 weeks at about 6–7 months of age. Contrary to this observation, guinea fowls have been reported to lay eggs during the dry season (September to April) in Botswana (Moreki and Seabo, 2012). Egg incubation were mostly natural and lasted from 26 to 29 days while the duration varied from 26 to 28 days according to some authors (Obun, 2004; Sanfo et al., 2007). This incubation period also varied from 27 to 28 days in Ivory Coast (Kouassi et al., 2019) and Bangladesh (Khairunnesa et al., 2016). This difference in duration can be attributed to the climatic conditions which differ from one country to another thus affecting embryonic development. The average hatchability rate was 74%. This hatching rate was relatively similar to earlier report in Benin (Boko et al., 2012), but higher than what was observed in Zimbabwe (63.8%) as documented by Zvakare et al. (2018). The average weight of guinea fowl obtained (1.3 kg) was lower than that obtained by Ogah (2013) in Nigeria, but higher than that reported in Ghana (Agbolosu et al., 2015; Brown et al., 2017). This average weight varies by region. These variations in weight may be due to the environmental conditions which differ from one region to another and which can be favorable or not to the good growth of guinea fowls. In rural areas, a mortality rate of 10% was observed one week after hatching. This mortality rate could be as high as 22% at 3 months of age. To limit these mortalities, farmers used the bark and leaves of certain locally available plants which are macerated and included in the drinking water of guinea fowl. Some of these plants materials employed by farmers during the survey include *Azadirachta indica* and *Khaya senegalensis*. Old practices relating to the use of traditional medicine are still relevant because of the low income of farmers and their distance from urban centres. Nevertheless, the use of traditional medication still has its drawback in most cases due to non-precise diagnosis and medication dosage (Hien et al., 2005). Therefore, it would be necessary to verify the effectiveness of these ethno-veterinary plants in order to validate for a better recommendation (Nyoni and Masika, 2012).

About the characterization of guinea fowl farming system, the results of the present study made it possible to identify four categories of guinea fowl farmers in Benin which differed based on location, sex, level of education, activity carried out and type of incubation. In Alibori region, where guinea fowl production in general constituted the primary occupation of respondents, followed by crop production, women were moderately involved in guinea fowl rearing. This result is explained by the involvement of more men in large ruminants production, which they believe was more profitable. In this region, the incubation of eggs was almost natural through the involvement of mother hens, ducks and turkeys. On the other hand, women were fairly involved in guinea fowl farming in the region of Atacora where agriculture was the dominant activity but associated with guinea fowl production. Guinea fowl farming was mainly engaged in by men in Benin unlike the case of Zimbabwe (Ndiweni, 2013) where women were more involved in this sector. Individuals with a high level of education were involved in guinea fowl rearing as a secondary activity. These results suggest that guinea fowl production was mostly done by illiterates, who had more empirical experience in the field as also reported by Kwesisi et al. (2015). In terms of comparison of the four groups of guinea fowl farmers, it can be deduced that cluster 3 showed the best performance. It is made up of mostly young farmers between 25 and 50 years of age with middle experience in guinea fowl breeding. Although most of the respondents in this group are represented in almost all regions, they are best found in the Borgou, Couffo and Mono regions. These guinea fowl farmers mostly adopt a semi-intensive breeding system and use artificial incubation to hatch their eggs better than others clusters. Cluster 4 farmers, who were better represented in Atlantique, Collines and Zou regions, took the second place based on these variables (experience in guinea fowl breeding, number of eggs at start-up, farming system, incubation, additives in feed and water). The farmers in cluster 2 took the third position and those in cluster 1 who were relatively women occupy the last place in this classification. Any capacity building and support program should be aimed at these clusters of farmers in order to get more women involved in guinea fowl farming and ultimately increase the productivity of the species. However, in Botswana, Moreki et al. (2010) reported that women (67.24%) were mainly beneficiaries of guinea fowl projects. This situation, although deliberately targeted women, demonstrated that women can also raise guinea fowl. This last cluster (cluster 1) had more illiterate farmers than all other groups. This is part of the reason for their poor performance. Nevertheless, this high rate of illiteracy, which is not peculiar to guinea fowl production, is a potential disadvantage for large-scale production of guinea fowl because of its negative influence on the adoption of new technologies (Issaka and Yeboah, 2016; Mugumaarhahama et al., 2016).

## 5 Conclusions

Guinea fowl farming is an important enterprise for the rural population, ranging from its contribution to food security to the fight against poverty through animal protein and the income that guinea fowl meat and eggs provide. The commonest management system practiced was the semi-intensive system that was largely dominated by crop farmers, mostly men and without any formal education. The constraints to guinea fowl production can be overcome by training farmers and providing them with adequate technical information translated into the most representative local languages with highly illustrative images to facilitate better understanding to the farmers due to the high level of illiteracy. Also, formulation of feed based on available local by-products and the use of drinking water for the animals with an emphasis on the inclusion of products likely to strengthen the resilience of these birds to climate variability is expedient. For a better optimization of the potential of this birds species, it is mandatory to have a deeper knowledge on their phenotypic and molecular characterization.

## Declarations

### Author contribution statement

Boko Michel Orounladji: Conceived and designed the experiments; Performed the experiments; Analyzed and interpreted the data; Contributed reagents, materials, analysis tools or data; Wrote the paper.

Folasade O. Oke: Analyzed and interpreted the data; Wrote the paper.

Koffi Tozo: Conceived and designed the experiments; Contributed reagents, materials, analysis tools or data; Wrote the paper.

Christophe A. A. M. Chrysostome: Conceived and designed the experiments; Contributed reagents, materials, analysis tools or data; Wrote the paper.

### Funding statement

This work was supported by 10.13039/100004421World Bank. Grant number: 10.13039/501100001450IDA 5424 LOME.

### Data availability statement

Data included in article/supplementary material/referenced in article.

### Declaration of interests statement

The authors declare no conflict of interest.

### Additional information

No additional information is available for this paper.

## References

[bib1] Abdul-Rahman I.I., Adu Y.E. (2017). http://www.lrrd.org/lrrd29/4/iddr29072.html.

[bib2] Agbolosu A.A., Ahunu B.K., Aboagye G.S., Naazie A., Kayang B.B. (2015). Variation in some qualitative traits of the indigenous Guinea fowl in Northern Ghana. Glob. J. Anim. Sci. Res..

[bib3] Audigier V., Husson F., Josse J. (2017). MIMCA: multiple imputation for categorical variables with multiple correspondence analysis. Stat. Comput..

[bib4] Avornyo F.K., Salifu S., Panyan E.K., Al-Hassan B.I., Ahiagbe M., Yeboah F. (2016). http://www.lrrd.org/lrrd28/8/avor28134.html.

[bib5] Boko K.C., Kpodekon T.M., Dahouda M., Marlier D., Mainil J.G. (2012). Contraintes techniques et sanitaires de la production traditionnelle de pintade en Afrique subsaharienne. Ann. Med. Vet..

[bib6] Brown M.M., Alenyorege B., Teye G.A., Roessler R. (2017). Phenotypic diversity, major genes and production potential of local chickens and Guinea fowl in Tamale, northern Ghana. AJAS (Asian-Australas. J. Anim. Sci.).

[bib7] Dahouda M. (2003).

[bib8] Fotsa J.C. (2008).

[bib9] Gawande S.S., Kalita N., Barua N., Saharia K.K. (2007). Elevage du poulet local en milieu rural d’Assam (Inde). Aviculture Familiale.

[bib10] Hien O.C., Diarra B., Drabo Y., Boly H., Sawadogo L. (2005). Pratiques de l’aviculture traditionnelle par les différents groupes ethniques de la région des Cascades au Burkina Faso. Agron. Afr..

[bib11] Houndonougbo P.V., Chrysostome C.A.A.M., Mota R.R., Hammami H., Bindelle J., Gengler N. (2017). Phenotypic, socio-economic and growth features of Guinea fowls raised under different village systems in West Africa. Afr. J. Agric. Res..

[bib12] INSAE (Institut National de la Statistique et de l'Analyse Economique) (2015). https://urlzs.com/GN61s.

[bib13] Issaka B.Y., Yeboah R.N. (2016). Socio-economic attributes of Guinea fowl production in two districts in Northern Ghana. Afr. J. Agric. Res..

[bib14] Johnston L.G., Sabin K. (2010). Echantillonnage déterminé selon les répondants pour les populations difficiles à joindre. Methodol. Innovat. Online.

[bib15] Khairunnesa M., Das S.C., Khatun A. (2016). Hatching and growth performances of Guinea fowl under intensive management system. Progr. Agric..

[bib16] Kone G.A., Kouassi G.F., Kouakou N.D.V., Kouba M. (2018). Diagnostic of Guinea fowl (*Numida meleagris*) farming in Ivory Coast. Poultry Sci..

[bib17] Kouassi G.F., Koné G.A., Good M., Kouba M. (2019). Factors impacting Guinea fowl (*Numida meleagris*) production in Ivory Coast. J. Appl. Poultry Res..

[bib18] Kusina N.T., Saina H., Kusina J.F., Lebel S. (2012). An insight into Guinea fowl rearing practices and productivity by Guinea fowl keepers in Zimbabwe. Afr. J. Agric. Res..

[bib19] Kwesisi V., Oloko M., Ommeh S. (2015). Value chain and market perfomance for poultry in Kenya: case of Guinea fowls & quails. Int. J. Manag. Commerce Innovat..

[bib20] Lombo Y., Tona K., Talaki E., Bonfoh B. (2018). Effet de l’alimentation sur la croissance des pintadeaux au Nord du Togo. Int. J. Biol. Chem. Sci..

[bib21] Lopez-Pedrouso M., Cantalapiedra J., Munekata P.E.S., Barba F.J., Lorenzo J.M., Franco D., Lorenzo J., Munekata P., Barba F., Toldrá F. (2019). More than Beef, Pork and Chicken – the Production, Processing, and Quality Traits of Other Sources of Meat for Human Diet.

[bib22] Mishra S., Kataria J.M., Sah R.L., Verma K.C., Mihra J.P. (2001). Studies on the pathogenicity of Newcastle disease virus isolate in Guinea fowl. Trop. Anim. Health Prod..

[bib23] Moreki J.C., Seabo D. (2012). Guinea fowl production in Botswana. J. World Poult. Res..

[bib24] Moreki J.C., Thutwa M., Ntesang K., Koloka O., Ipatleng T. (2010). http://www.lrrd.org/lrrd22/11/more22210.htm.

[bib25] Moussa Amadou B., Idi A., Benabdeljelil K. (2010). Aviculture familiale rurale au Niger: alimentation et performances zootechniques. Communications en Aviculture Familiale.

[bib26] Mugumaarhahama Y., Ayagirwe R.B.B., Mutwedu V.B., Sadiki J.M., Baenyi P., Mushagalusa A.C., Bisimwa E.B. (2016). http://www.lrrd.org/lrrd28/1/mugu28007.html.

[bib27] Ndiweni N.J. (2013). Prudent poultry farming as a source of livelihood and food security in a changing climate: the case of Zhombe communal lands, Zimbabwe. Int. J. Sci. Res. Publ..

[bib28] Nyoni N.M.B., Masika P.J. (2012). Village chicken production practices in the Amatola basin of the Eastern Cape Province, South Africa. Afr. J. Agric. Res..

[bib29] Obun C.O. (2004). Hatching and brooding of Guinea fowl (*Numida meleagris galeata pellas*) egg using local hen. Global J. Agric. Sci..

[bib30] Ogah D.M. (2013). Variability in body shape characters in an indigenous Guinea fowl (*Numida meleagris* L.). Slovak J. Anim. Sci..

[bib31] R Core Team (2019). https://www.R-project.org/.

[bib32] Sanfo R., Boly H., Sawadogo L., Brian O. (2009). Eléments d’analyse de l’élevage villageois de la pintade locale (*Numida meleagris*) dans le Plateau Central du Burkina Faso. Rev. Afr. Santé Prod. Anim..

[bib33] Sanfo R., Boly H., Sawadogo L., Ogle B. (2007). Caractéristique de l’élevage villageois de pintade locale (*Numida meleagris*) au centre du Burkina Faso. Tropicultura.

[bib34] Sayila A. (2009). Guinea fowl farming becomes popular in Botswana. World Poultrymeat.

[bib35] Soara A.E., Talaki E., Tona K. (2020). Characteristics of indigenous Guinea fowl (*Numida meleagris*) family poultry production in northern Togo. Trop. Anim. Health Prod..

[bib36] Traore F.G., Traore A., Bayala B., Dayo G.K., Tapsoba A.S., Soudre A., Sanou M., Tindano K., Tamboura H.H. (2018). Characterization and typology of Guinea fowl (*Numida meleagris*) farming systems in Burkina Faso. Int. J. Adv. Res..

[bib37] Zvakare P., Mugabe P.H., Mutibvu T. (2018). Guinea fowl (*Numida meleagris*) production by small-holder farmers in Zimbabwe. Trop. Anim. Health Prod..

